# Supplementary material for: A leg model based on anatomical landmarks to study 3D joint kinematics of walking in Drosophila melanogaster
Source: Front Bioeng Biotechnol. 2024 Jun 26;12:1357598. doi: 10.3389/fbioe.2024.1357598 (PMC11233710; doi:10.3389/fbioe.2024.1357598)
Supplement: Supplementary file 6 [file DataSheet1.docx]

Supplementary Material

A leg model based on anatomical landmarks to study 3D joint kinematics of walking in *Drosophila melanogaster*

Moritz Haustein^1^, Alexander Blanke^2^, Till Bockemühl^1‡^, Ansgar Büschges^1‡*^

^1^Institut of Zoology, Biocenter Cologne, University of Cologne, Cologne, Germany

^2^ Bonn Institute for Organismic Biology (BIOB), Section 2: Animal Biodiversity, University of Bonn, Bonn, Germany

^‡^Shared senior authorship

*** Correspondence:** Ansgar Büschges: [ansgar.bueschges@uni-koeln.de](mailto:ansgar.bueschges@uni-koeln.de)

# Supplementary Figures


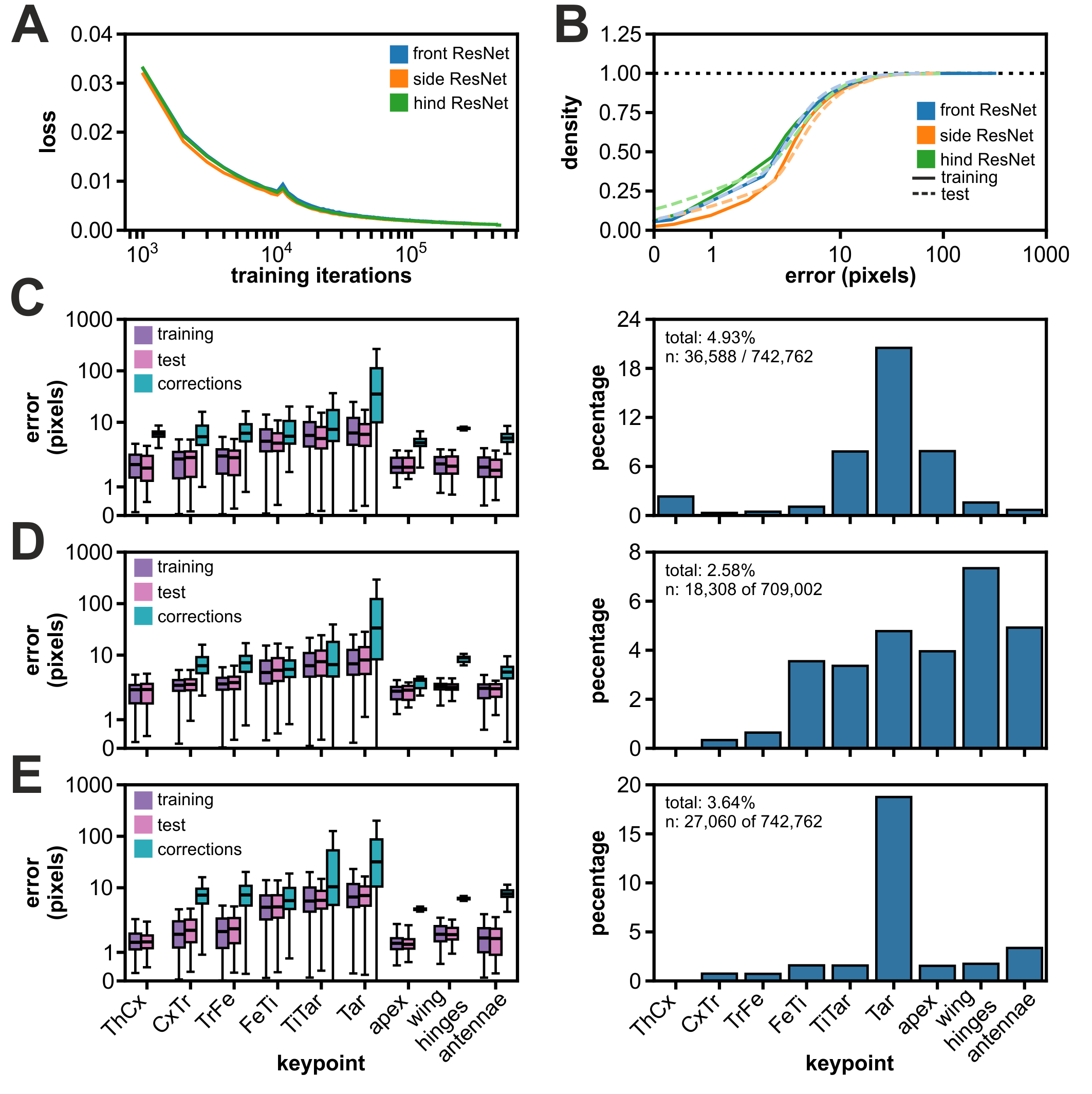


**Supplemental Figure 1. Evaluation of DeepLabCut (DLC) performance.** (A) DLC training curves for the independent front, side, and hind ResNet-50 networks used to track body keypoints in different camera views. (B) Cumulative error distribution of the training (solid lines) and test, i.e. out-of-sample, (dashed lines) error for the front, side, and hind ResNet-50 networks after training was completed. (C-E) Localization error of individual tracked body keypoints for the front (C), side (D), and hind (E) ResNet-50 networks. Left panels show the error distribution as boxplots for the training, test, and manual correction datasets. Note that the larger error in the correction dataset can be expected insofar as the correction dataset consisted of obvious false detections of the networks. Outliers were omitted for clarity. Right panels represent the frequencies of required manual corrections of individual keypoints based on visually inspection of all experimental videos.


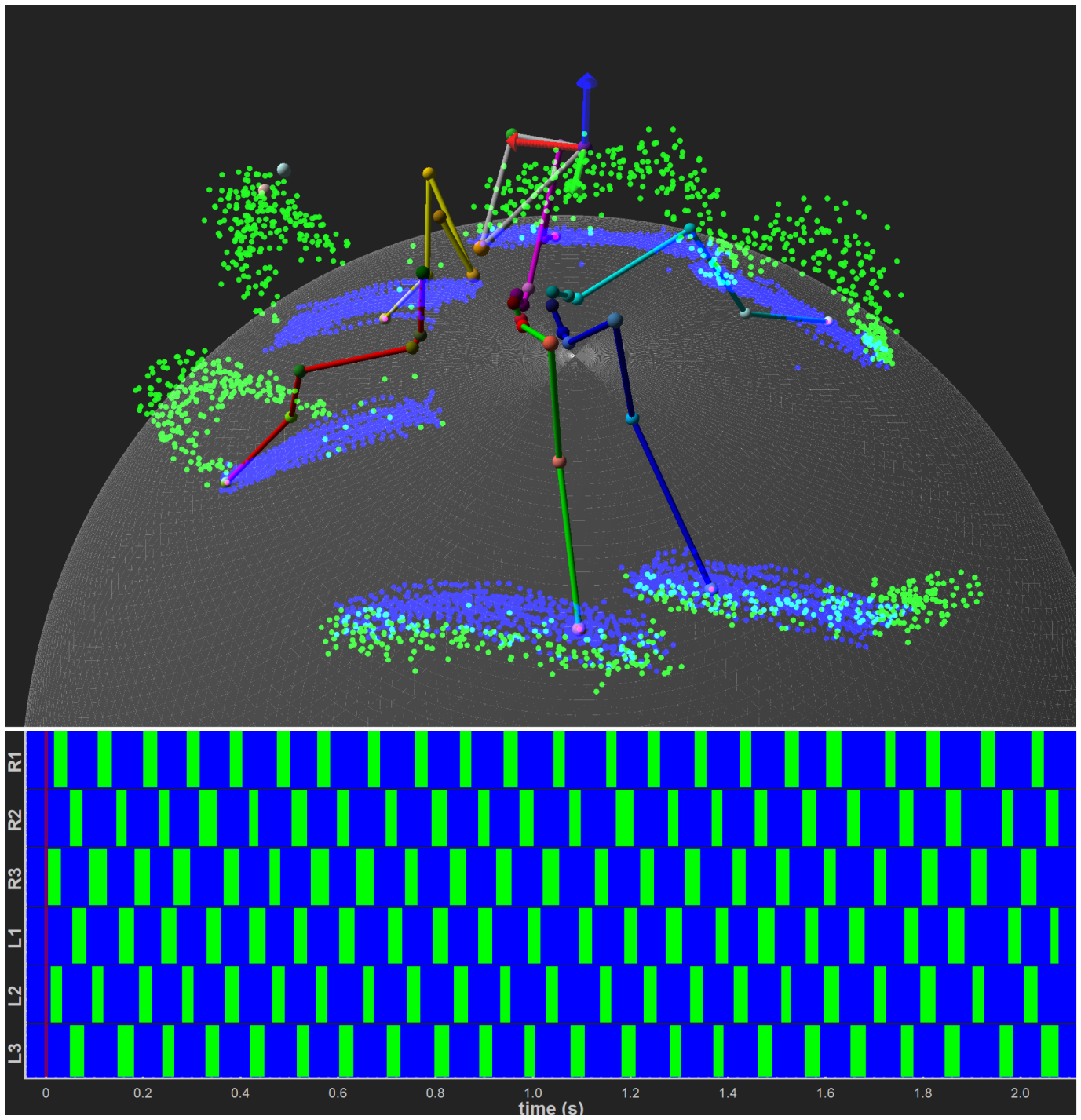


**Supplemental Figure 2. Example of swing and stance phase detection.** The upper panel shows 3D- reconstructed tarsus tips positions of a walking sequence from a fly. Blue dots represent positions when the tarsus is on the ground (stance phase), while green dots represents positions when the tarsus is lifted off the ground (swing phase). The lower panel shows the resulting footfall pattern for the complete walking sequence.


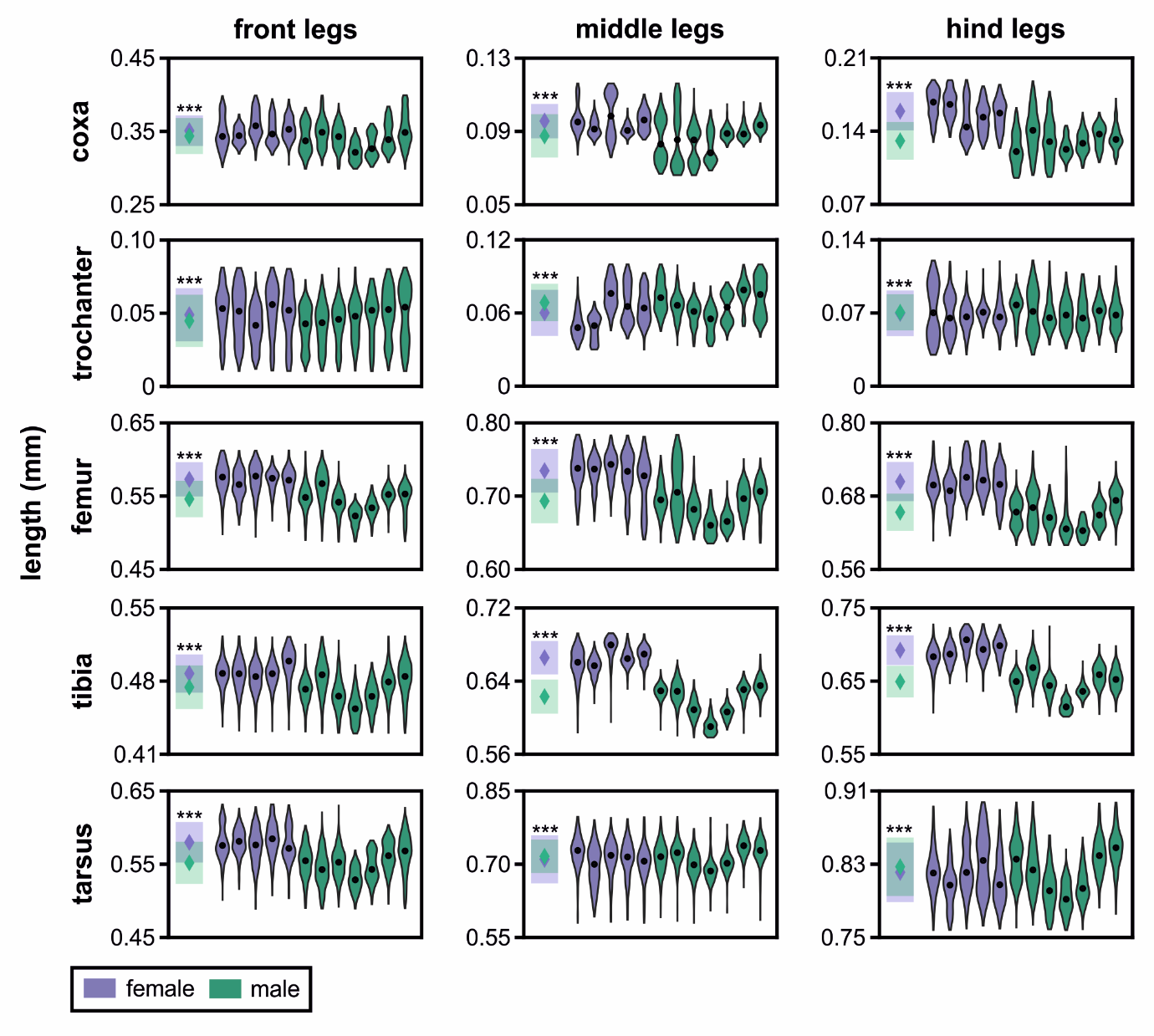


**Supplemental Figure 3**. **Measured lengths of leg segments for males and females.** Based on the leg segment lengths of motion captured data set. For this, the distance between two adjacent joints or the tarsus tip were calculated for each motion captured leg posture, i.e. each video frame. Leg segments for left and right legs were pooled. Colored diamonds and boxes represent the mean ± SD for all flies (n = 6530/4724 female/male), respectively. Independent t-test results are displayed as: n.s. (not significant): p > 0.05; *: p < 0.05; **: p < 0.01; ***: p < 0.001. Note that, although we found a trend that leg segments are larger in females than in males, the mean lengths did not differ considerable for most leg segments. Violin plots show the lengths for motion captured leg segments for each fly (from left to right: n = 1364, 932, 1184, 814, 430, 1368, 1100, 968, 462, 442, 528, 1662). Black circles in the violin plots represent the median leg segment length for each fly which was eventually used to define the lengths in the kinematic model for each fly. For clarity, extreme values below 1 % and above 99% percentiles were excluded for individual flies in the violin plots.

**
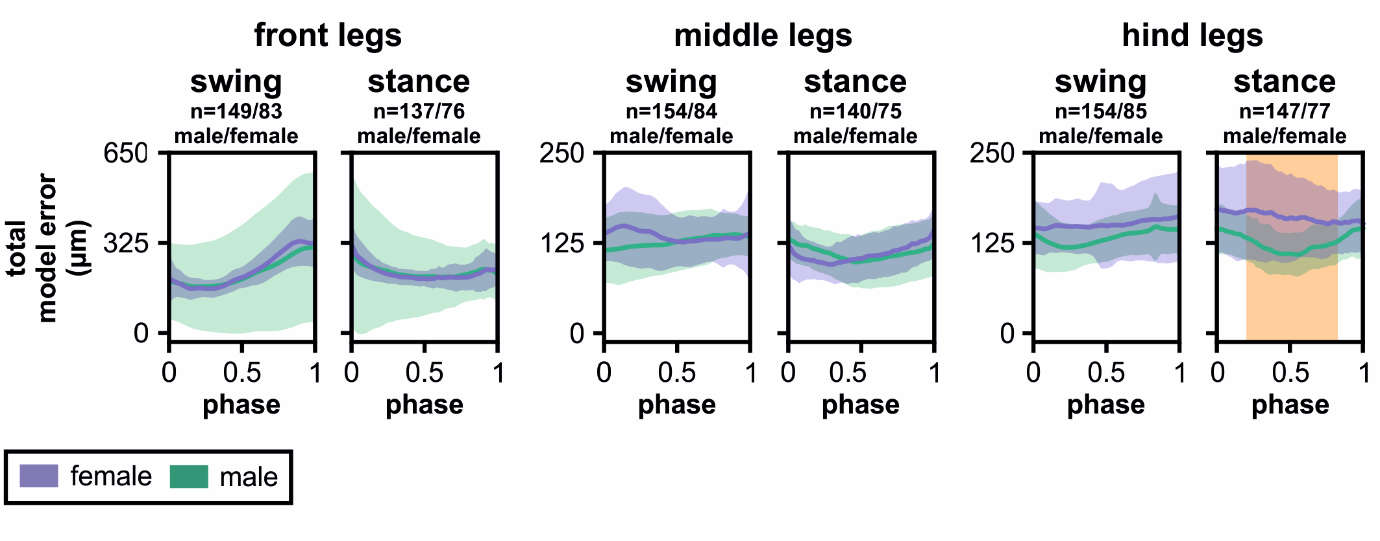
**

**Supplemental Figure 4.** **The total error of the kinematic leg model is comparable between female and male flies.** The time course of the total mean error for front, middle, and hind legs during swing and stance phases. The reference model DOF configuration was used for all leg pairs, but the kinematic model for the front legs was equipped with an extra TrFe-roll DOF. Colored lines and areas represent the mean ± SD for females (purple, N=5) and males (green, N=7). Orange boxes indicate results of cluster-based permutation analysis, i.e. identified clusters with p-values < 0.05.


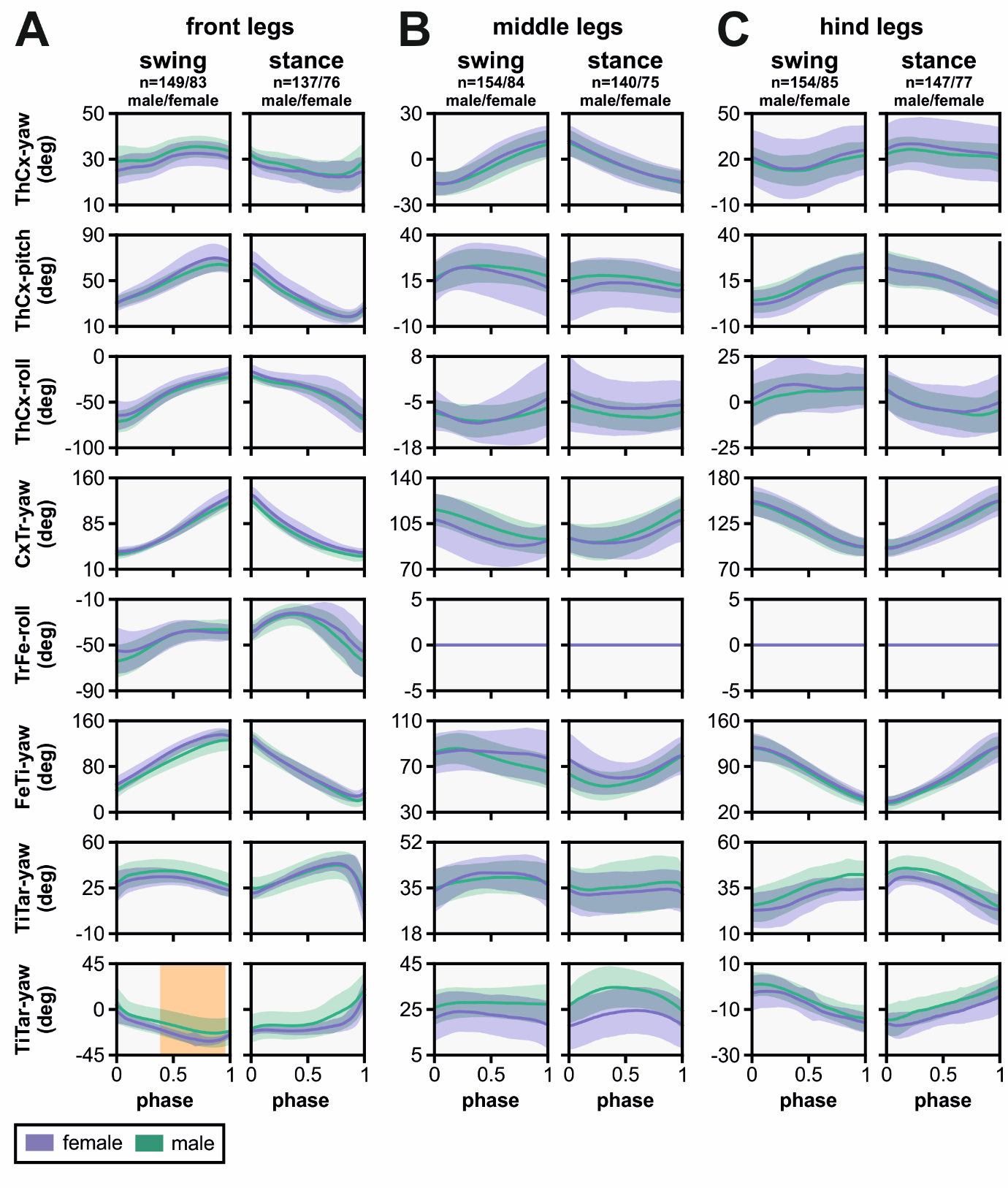


**Supplemental Figure 5.** **Female and male flies have comparable joint angle kinematics.** The joint angle time courses are shown in relation to the normalized swing and stance phase for front (A), middle (B), and hind (C) legs. Angles were calculated in relation to the initial posture of the model, except for CxTr-yaw and FeTI-yaw DOFs. These were post-processed to show the relationship between the linked segments: 0° indicates a complete overlap of both segments, i.e. maximally flexed, while 180° indicates that both segments are co-linear, i.e. maximally extended. Lines and areas represent the mean ± SD for females (purple, N=5) and males (green, N=7). Orange boxes indicate results of cluster-based permutation analysis, i.e. identified clusters with p-values < 0.05.
